# A New Method of Using Politzer’s Apparatus

**Published:** 1894-06

**Authors:** 


A NEW METHOD OF USING POLITZER'S APPARATUS.—Those who have had occasion to use the Politzer bag to inflate the middle ear are well aware how difficult it is to cause the patient to swallow at the proper moment.

The general custom is to ask the patient to sound certain vowels or to swallow a mouthful of water, because in uttering these sounds, and during the act of deglutition, the soft palate is applied to the posterior wall of the pharynx. The fact remains, however, that the naso-pharyngeal cavity is only partially excluded by these means, and then only for a most brief—frequently too brief—period.

As the Politzer method of clearing the Eustachian tube is frequently of material importance, it may not be amiss to call attention to a novel and more simple way of preventing the air insufflated into the nose from escaping through the pharynx instead of passing into the tympanic cavity, suggested by the *Medical Press and Circular* as follows:

As the clearing of the Eustachian tube is now very much in vogue, and moreover in many instances very essential to the treatment of certain diseases of the auditory apparatus, it may be well to mention a novel and more simple method of attaining the object in view as arrived at by Doctor Boydan, in the *Medical Times and Hospital Gazette.* This gentleman suggests that the patient be directed to take a deep inspiration and then slowly blow out the air through a small aperture between the closed lips.

The rationale of procedure is that, so long as the patient blows, the velum palati remains in contact with the posterior wall of the pharynx, and Politzerization can be performed without the slightest difficulty.—*Medical Age.*

